# Vancomycin Use in Children and Neonates across Three Decades: A Bibliometric Analysis of the Top-Cited Articles

**DOI:** 10.3390/pathogens10101343

**Published:** 2021-10-18

**Authors:** Chiara Minotti, Elisa Barbieri, Carlo Giaquinto, Daniele Donà

**Affiliations:** Division of Pediatric Infectious Diseases, Department of Women’s and Children’s Health, University of Padova, 35100 Padova, Italy; elisa.barbieri@unipd.it (E.B.); carlo.giaquinto@unipd.it (C.G.); daniele.dona@unipd.it (D.D.)

**Keywords:** vancomycin, bibliometric analysis, top-cited articles, pediatric infectious diseases, children, neonates

## Abstract

Vancomycin is frequently prescribed in pediatrics, especially in intensive care unit settings, to treat Gram-positive bacterial infections. This work aims to collect the top-cited articles of pediatric and infectious diseases areas to gather the current evidence and gaps of knowledge on the use of vancomycin in these populations. The most relevant journals reported in the “pediatrics” and “infectious diseases” categories of the 2019 edition of Journal Citation Reports were browsed. Articles with more than 30 citations and published over the last three decades were collected. A bibliometric analysis was performed and 115 articles were retrieved. They were published in 21 journals, with a median impact factor of 4.6 (IQR 2.9–5.4). Sixty-eight of them (59.1%) belonged to “infectious diseases” journals. The most relevant topic was “bloodstream/complicated/invasive infections”, followed by “antibiotic resistance/MRSA treatment”. As for population distribution, 27 articles were on children only and 27 on neonates, most of which were from intensive care unit (ICU) settings. The current literature mainly deals with vancomycin as a treatment for severe infections and antibiotic resistance, especially in neonatal ICU settings. Lately, attention to new dosing strategies in the neonatal and pediatric population has become a sensible topic.

## 1. Introduction

Vancomycin is one of the most frequently prescribed glycopeptides, especially in children and newborns in intensive care unit (ICU) settings for the treatment of Gram-positive bacterial infections by coagulase-negative Staphylococci (CoNS), Enterococci spp, methicillin-resistant *Staphylococcus aureus* (MRSA), and *C. difficile*. It inhibits cell wall synthesis by binding to the D-Ala-D-Ala terminal of the peptide chain and has a volume of distribution of 0.4–1 L/kg [1]. There is significant variability in protein binding, which is believed to be up to 50% [2]. Its bactericidal activity is related to the area under the curve (AUC) and minimal inhibitory concentration (MIC) ratio (AUC/MIC ratio), which, as stated in the literature, has to be higher than 400 to guarantee standard efficacy [1]. Serum levels monitoring is recommended, with a target trough concentration goal of 15–20 µg/mL for severe infections. Based on these pharmacodynamics data, continuous infusion of vancomycin has been proposed for severe infections, guaranteeing higher steady-state concentrations. However, there is little consensus on choosing the optimal dosing regimen and administration schedule (intermittent vs. continuous infusion) in children and even more in neonates, especially in preterm and extremely preterm patients. 

Another concern is the emergent antibiotic resistance issues, with the appearance of vancomycin-intermediate strains in methicillin-resistant *S. aureus* (MRSA) infections after the exposure to prolonged vancomycin therapy, due to the selection of resistant subpopulations under the pressure of antimicrobial exposure [3]. More recently, increasing MICs and decreased susceptibility have been reported for CoNS as well, for the same reason, with subsequent treatment failures [4,5].

The pediatric infectious diseases consultant is often involved in choosing the most appropriate antibiotic therapy in complex cases and implementing antibiotic stewardship programs. Pediatric infectious diseases as a clinical sub-specialty is accessible by pediatricians and infectious diseases specialists who share the management of severe infections and choice of therapy in pediatric patients and, therefore, require extensive knowledge and experience. Most scientific papers address mainly or exclusively the adult population, and evidence on children or neonates is often scattered in journals of both pediatric and infectious diseases areas. This might increase the difficulty in finding the most relevant sources. Their collection might help trainees and consultants improve their knowledge and monitor what is new in this field.

This bibliometric analysis aims to collect the top-cited articles in the last three decades related to vancomycin and its use in the pediatric and neonatal population. Secondary aims are to identify the most relevant scientific journals dealing with pediatric infectious diseases and summarize the most important topics regarding new dosing regimens, especially for neonates, in order to underline relevant gaps of knowledge to date. 

## 2. Materials and Methods 

The 2019 edition of *Journal Citation Reports (JCR): Science Edition* was browsed by category to identify highly indexed journals. All the journals of the first quartile belonging to the “pediatrics” and “infectious diseases” categories were considered in order to include those with the highest impact factor (IF). Overall, 55 journals were searched for the study. Thirty-two belonged to the “pediatrics” category, and the remaining 23 to “infectious diseases”. A complete list of the journals, ranked by their IF is provided in the Supplementary Materials (Supplementary Tables S2 and S3).

The *Web of Science–Science Citation Index Expanded* database was searched on September 10th, 2021. The inclusion criteria were the English language, a minimum of 30 citations, publication dates ranging from January 1990 to September 2021, the pertinence with vancomycin in general, and its use in children and neonates, assessed by the authors, excluding all studies only addressing the adult population. The articles meeting the inclusion criteria were ranked by citation number. If two or more articles were equally cited, the IF of the journal was considered the discriminating factor. A top-cited list was produced.

The articles were further classified according to article type, topic, population, ICU vs. non-ICU setting, and country of origin. The topics were classified by the Authors, starting from the keywords of the articles, and were included in the following categories: adverse reactions, MIC interpretative criteria, ototoxicity, pharmacokinetics and pharmacodynamics, antibiotic stewardship, healthcare epidemiology, nephrotoxicity, *C. difficile* treatment, dosing strategies/intermittent vs. continuous infusion, state of the art, antibiotic resistance/MRSA treatment, bloodstream/complicated/invasive infection treatment. 

Among the evaluated parameters in our bibliometric analysis, we made a gender description of the first and last authors. The *PubMed/MEDLINE* database was searched for further information on these topics. Continuous data were reported as median and inter-quartile range (IQR), while non-continuous data were reported as numbers.

## 3. Results

### 3.1. Scientific Journals

One hundred and fifteen top-cited articles were retrieved in total, published in 21/55 journals, with a median IF of 4.6 (IQR 2.9–5.4). The excluded articles only addressing adults were 227. The journal with the highest IF was *Lancet Infectious Diseases* (IF 24.446), and the one with the lowest was *Pediatric Nephrology* (IF 2.676). Table 1 shows the main features of the manuscripts.

Sixty-eight articles (59.1%) were published in “infectious diseases” category journals. 

The median number of top-cited articles per journal was 2 (IQR 1.5–3), and the journal with the highest number of top-cited articles was “clinical infectious diseases” with 46 manuscripts. As for gender description of first and last authors, male authors were predominant (81 vs. 34 and 96 vs. 19, respectively). Only in the pediatrics area subgroup, was there an almost equal number of male and female first authors (Table 1).

### 3.2. Top-Cited Articles

The median number of citations was 94 (IQR 51–178), ranging from 30 to 2796. The complete list of the top-cited articles in the “infectious diseases” and “pediatrics” categories is displayed in Supplementary Materials (Supplementary Table S1). 

As for article type, 57 top-cited articles were original articles, 38 were guidelines and/or Reviews of the literature, and only nine papers were clinical trials. 

**Figure 1:**
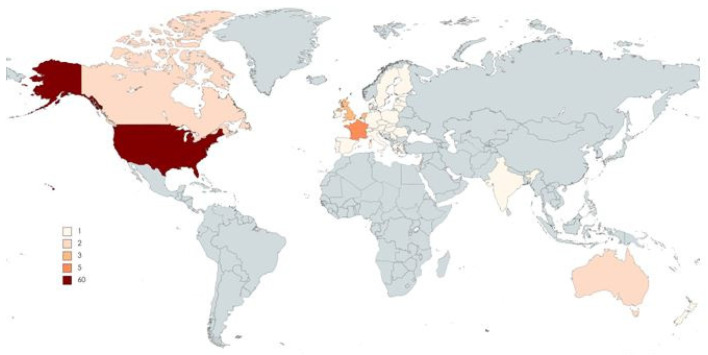
Top-cited articles by country/region of origin.

As shown in Figure 1, the most productive country of origin was the USA, with 71 manuscripts. However, 12 papers were multicenter, involving more than two countries.

Topics are displayed in detail in Table 2. The most treated topic was “bloodstream/complicated/invasive infections”, with 30 articles, followed by “antibiotic resistance/MRSA treatment”, with 27 papers, and “state of the art” papers (13 papers). Eleven articles were about “dosing strategies/continuous vs. intermittent infusion” and ten about *C. difficile* treatment (Table 2). 

As regards continuous versus intermittent infusion, most studies addressed the neonatal population. Some presented simplified schedules for continuous infusion in neonates, according to body weight and serum creatinine, that led to adequate serum vancomycin levels and a good efficacy profile, also reducing prescription error rates in this population (Table 2) [58,59,60,61,62,63,64,65,66,67,68,69,70,71,72,73,74,75,76,77,78,79,80,81,82,83,84,85,86,87,88,89,90,91,92,93,94,95,96,97,98,99,100,101,102,103,104,105,106,107,108,109,110,111,112,113,114,115,116,117,118,119,120]. 

Concerning the treatment of *C. difficile* infection, the oral administration of vancomycin is recommended in children (10 mg/kg/dose four times a day for ten days) for the initial episode, severe or non-severe, with a slow décalage in case of recurrence [104,105,106,107,108,109,110,111,112,113]. 

Other topics were “nephrotoxicity”, with six articles, “healthcare epidemiology” and “antibiotic stewardship” (four papers each). Papers dealing with nephrotoxicity mostly identified high vancomycin trough levels (>15 mg/L) and concomitant furosemide use as risk factors for the development of kidney injury [69,70].

Studies on epidemiology mainly focused on hospital-acquired and MRSA infections in Neonatal Intensive Care Units (NICUs), antibiotic exposure, and inflammatory bowel disease development [9,10,11,12]. Papers reporting antibiotic stewardship experiences were from NICU and Pediatric Critical Care units, showing the widespread use of vancomycin, considered inappropriate in large proportion in critically ill children and neonates [114,115]. 

The least reported topics were respectively “pharmacokinetics and pharmacodynamics” (PK/PD) and “adverse reactions” (three papers each), “ototoxicity”, in relation to hearing screening in newborns, and “MIC interpretative criteria”, with two articles each (Table 2).

In particular, only one of the retrieved articles on PK/PD was specific for the pediatric population, while the other two dealt with PK/PD features in general and about drug penetration in biofilm [1,14]. The most useful parameters for the evaluation of vancomycin PK/PD correlation are the AUC and MIC. As reported by Rybak et al., an AUC/MIC ratio higher than 400 is related to a plasma trough level above 15 µg/mL, assuming 1 mg/L MIC or less [1]. Model studies have reported that the current empiric recommended vancomycin dose in children of 40 mg/kg/day would unlikely achieve the recommended pharmacodynamic target of AUC 24/MIC >400 in case of methicillin-resistant *S. aureus* (MRSA) with MIC of 1.0 µg/mL or greater, proposing an increase of the dose to 60 mg/kg/day [120,121].

Twenty-seven papers dealt with neonates, 18 of which in NICU contexts, dealing with vancomycin for the treatment of neonatal infections, especially late-onset sepsis due to Gram-positive bacteria, mainly CoNS and MRSA [11]. Major topics were antibiotic resistance and dosing strategies, which reflect the main issues in this population to date. Ototoxicity and nephrotoxicity were addressed as the most important aspects to consider in the follow-up of neonates after vancomycin treatment [66,73]. The recommended intravenous dose for treating sepsis or severe infections in neonates is 10–15 mg/kg, 15 mg/kg for central nervous system infections, with varying intervals according to gestational and postnatal age (once every 18/12/8 h accordingly). According to most studies, for infants older than one month and children up to 12 years with a normal renal function, the advised intravenous daily dose is 60 mg/kg in four divided doses. During treatment of serious infections, including those related to MRSA, trough levels should be monitored, with a target concentration goal of 15–20 μg/mL [120].

Key points on vancomycin use in pediatric patients emerging from our analysis, including what is already known and gaps of knowledge for the development of future studies, are summarized in Table 3. 

## 4. Discussion

To our knowledge, this is the first bibliometric analysis with a special interest in vancomycin in the pediatric and neonatal populations. 

**Figure 2:**
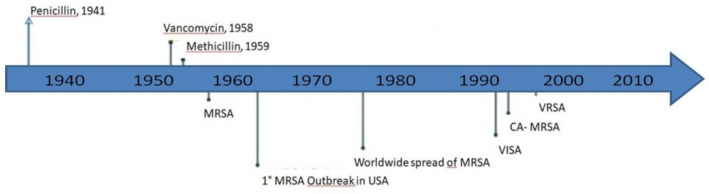
*S. aureus* drug resistance development through the years (adapted from McGuinnes et al. [5]). MRSA: Methicillin-resistant *S. aureus*; VISA: vancomycin-intermediate *S. aureus*; CA: community-acquired; VRSA: vancomycin-resistant *S. aureus*.

We chose evaluative bibliometrics and the method of citation analysis to evaluate research performance in this particular field and to highlight gaps of knowledge for the development of future studies. We decided to consider the last three decades as a definite search period, because especially in those years vancomycin was extensively used, with a consequent selection pressure, determining the emergence of resistance [5,26] (Figure 2). 

Not surprisingly, the journal with the highest number of top-cited articles was in the infectious diseases and not the pediatric area, being ranked third among the journals of the same area. Among the top-ranked journals of the pediatric area, there were no pediatric infectious diseases sub-specialty journals. This may suggest that the search for the most relevant articles in pediatric infectious diseases should be performed not only among pediatric journals but mainly on the most impacted journals treating topics of infectious diseases. 

As for the article types, the second most cited, after original articles, were guidelines and/or reviews of the literature. According to the hierarchy provided by the Center for Evidence-Based Medicine, the latter presents the highest level of evidence [122]. They mostly were clinical practice guidelines by Infectious Diseases societies or dealt with current evidence about prescription and dosing in children and neonates. The most represented topics were antibiotic resistance, complicated infections, and *C. difficile* infections, correctly reflecting the main fields and emerging issues involving vancomycin and its use in pediatric patients. 

The two most treated topics in the top-cited articles were complicated infections and antibiotic resistance. Again, this is not surprising, thinking of the extensive use of vancomycin to treat invasive infections in critical patients, leading to antibiotic selection pressure after prolonged exposure, with an increased risk of treatment failure [3,4,28,29,30,31,32,33,34]. Selection pressure by indiscriminate use of vancomycin, linked to at least four genes (Van A-D), has led to the emergence of vancomycin-resistant *Enterococci* (VRE). As regards *S. aureus*, vancomycin-intermediate (VISA), and vancomycin-resistant *S. aureus* (VRSA) strains are described, together with the resistance of *S. epidermidis*, mainly linked to biofilm [3]. As highlighted by van Hal et al in a systematic review and meta-analysis, emerging data show that vancomycin may be less effective to treat serious MRSA infections with higher MICs, with treatment failure concerns. An association of high MICs and higher mortality rates in MRSA bloodstream infections has been demonstrated. [119]. Therefore, prospective studies are needed to assess if optimizing vancomycin treatment can improve outcomes without toxicity issues. This opens the chapter on second-line treatments for vancomycin treatment failures.

Among the 115 retrieved articles, 27 were about children as a selected population, dealing above all with the management of invasive infections and *C. difficile* infections. Two papers had the implementation of antibiotic stewardship programs as a primary focus, one of which in the context of a pediatric critical care unit [114,115]. This is indeed of paramount importance, considering the worldwide efforts to implement the appropriate use of antibiotics, especially in ICU settings. Vancomycin has undergone indiscriminate use for many years also in the pediatric population and the stewardship interventions aim at early therapy stop in the absence of microbiological isolates. 

Despite the widespread use of vancomycin since its introduction on the market, only three top-cited papers dealt with PK/PD and one only specifically on the pediatric population. PK/PD studies and RCTs are lacking for the neonatal population, especially for preterm and extremely preterm infants, as reported by the many reviews retrieved in this study. 

Dosing and safety in the pediatric and neonatal population are challenging, especially concerning continuous infusion, because of pharmacokinetics changes throughout the different ages. In general, ototoxicity and nephrotoxicity pathophysiological mechanisms are still unclear. The relation to dose exposure and treatment duration has not been proved, and focused studies are still lacking. 

One central question is still open regarding the administration of vancomycin by continuous versus intermittent infusion in children and neonates. Adult evidence suggests that continuous infusion of vancomycin decreases nephrotoxicity and the incidence of infusion-related adverse events, while also diminishing time to therapeutic concentrations and drug costs [123,124]. Studies on preterm neonates and patients under three years of age on this topic are few to date, and thus evidence is limited, with data from the adult population being not completely applicable. Despite a general lack of consensus, continuous infusion regimens are already used in clinical practice in many centers, especially in the UK. Prolonged or continuous infusions strategies of time-dependent antibiotics are elsewhere still considered on a case-to-case basis in the pediatric population, and available data seem to indicate a higher probability of reaching target trough levels in children, with reported good clinical outcomes and safety profile [61,62,65,121,125]. 

Regarding gender description, the highest number of female first authors belonged to the Pediatrics area and were almost as represented as their male counterparts. Nevertheless, we identified a gender gap in medical research. Male authors were predominant as both first and last authors in the Infectious Diseases area and generally more represented in senior authorship in both areas. We decided to include this description as we believe it could be an interesting bibliometric parameter to be evaluated, as an added value for the readers. Any assumption related to a minor involvement of women in research teams or a less frequent presence as senior faculty members cannot be made based on our data and goes beyond the focus of our research. 

Last, limitations to our study are intrinsic to the analysis type. Above all, the most recent articles may not have reached 30 citations due to a mere matter of time, and all relevant studies published in languages other than English may have been missed. 

## 5. Conclusions and Future Perspectives

To conclude, top-cited articles about vancomycin use in children and neonates were almost equally distributed among journals of the “infectious diseases” and “pediatrics” areas. The most productive journal was *Clinical Infectious Diseases*. The most treated topics were bloodstream or complicated infections and antibiotic resistance/MRSA treatment. The pediatric area is indeed a critical one in the field of antibiotic therapy. In the last three decades, though less reported, also the role of antibiotic stewardship and attention to new dosing strategies in the neonatal and pediatric population have become sensible topics, which need to be further explored. As a widely used drug, data from studies properly conducted in the pediatric population, and not derived from adult studies, are needed. Moreover, the efficacy and safety of higher doses due to increases in MIC should be studied. Among the least represented topics, PK/PD, antibiotic stewardship, and dosing and infusion strategies, especially in neonates, represent a critical gap of knowledge, with a florid literature pointing out a lack of studies in the pediatric population since at least 30 years. This underlines the need for randomized-controlled trials to evaluate the clinical impact, safety, and acceptability of continuous infusion of vancomycin compared to intermittent infusion as a main challenge in the neonatal and pediatric population.

## Figures and Tables

**Table 1:** Main features of the top-cited articles.

Journal Area	Infectious Diseases Papers	Pediatrics Papers	Grand Total
Grand Total	68	47	115
IF, Mean (IQR)	6.5 (4.5–6.4)	3.9 (2.7–3.9)	4.6 (IQR 2.9–5.4)
Time cited, Median (IQR)	143.5 (244.75–85.75)	56 (36–83)	94 (51–178)
Setting			
No-ICU	64	31	95
ICU setting	4	16	20
Population			
Children	7	20	27
Children; neonates	1		1
General/adults and children	56	4	50
Neonates	4	23	27
Gender of First Authors			
Female	12	22	34
Male	56	25	81
Gender of Last Authors			
Female	8	11	19
Male	60	36	96

IF: impact factor; IQR: interquartile range; ICU: Intensive Care Unit.

**Table 2:** Top-cited articles by Topic: main findings.

Topic	Infectious Diseases Papers	Pediatrics Papers	Grand Total	Main Findings	References
	68	47	115		
Adverse reactions	2	1	3	Usually mild reactions, good safety profile. Red man syndrome: infusion-related histamine-like reaction; nephrotoxicity and ototoxicity; rash, eosinophilia, thrombophlebitis, chills, fever, neutropenia, and thrombocytopenia.	[6,7,8]
Healthcare epidemiology	/	4	4	Vancomycin as first-line therapy for CoNS, Enterococci spp., MRSA and empiric therapy for LOS in NICUs with endemic MRSA; increasing reports of VISA and VRSA. Vancomycin exposure in childhood is associated with IBD development.	[9,10,11,12]
PK/PD	2	1	3	Time-dependent antibiotic. 25–50% protein-bound, mainly to albumin and immunoglobulins. Almost exclusively eliminated by the renal route via glomerular filtration and to some extent via active tubular secretion. Penetration in biofilm is not optimal. Neonates: Volume of distribution varies between 0.38 and 0.97 L/kg, and clearance varies between 0.63 mL/kg/min (0.038 L/kg/h) and 1.4 mL/kg/min (0.084 L/kg/h). The AUC/MIC value may be the pharmacodynamic parameter that best correlates with a successful outcome. An AUC/MIC ratio ≥400 has been identified as the optimal target for clinical effectiveness. Trough serum vancomycin concentrations for monitoring the effectiveness of vancomycin (renal excretion). The pharmacokinetics of vancomycin in neonates and young infants depends on weight and serum creatinine and showed a wide interindividual variability. Lack of PK studies on continuous infusion.	[1,13,14]
State of the art	11	2	13	Antibiotic of choice against serious Gram-positive infections, more than 50 years after its introduction. Increasing evidence suggests that it may be losing its clinical efficacy against serious MRSA infections with MICs at the higher end of the susceptibility range. Slowly bactericidal and characterized by suboptimal properties such as PK (requiring twice-daily dosing and serum level monitoring) and complex variable tissue penetration. The optimal dosing in critically ill patients remains a contentious issue.	[15,16,17,18,19,20,21,22,23,24,25,26,27]
Vancomycin and antibiotic resistance	25	2	27	In 1996 first reported strain of S. aureus with reduced susceptibility to vancomycin. Heteroresistant VRSA: strains of *S. aureus* that contain vancomycin-resistant subpopulations for which the MICs of vancomycin for the parent strain are 1–4 mg/mL. Vancomycin treatment failures reported with methicillin-resistant *S. aureus* displaying a MIC of 2 mg/L. Trough serum vancomycin concentrations must be maintained at 110 mg/L to avoid the development of resistance. VRE: several phenotypes, high-level resistance due to altered ligase (vanA and vanB genes on transposon/plasmid). Low-level resistance (MIC 8–32 mg/L) and high-level (MIC ≥ 64 mg/L). Selection pressure, HAI, and hospital outbreaks more frequent.	[28,29,30,31,32,33,34,35,36,37,38,39,40,41,42,43,44,45,46,47,48,49,50,51,52,53,54]
Vancomycin and dosing strategies/intermittent vs. continuous	3	8	11	Dosing optimisation is paramount to avoid resistanceand maximise the likelihood of achieving the therapeutic target. Adults: Continuous infusion in critically ill patients, to keep serum concentrations above a targeted MIC and for easier monitoring of drug concentrations and dosage adjustment. Target concentrations are obtained faster, with less variability in daily infused dose, though no difference in efficacy. Decreased risk of adverse reactions, including nephrotoxicity. Neonates, bacterial sepsis: prescription is challenging for high pharmacokinetic variability, lack of consensus on dosing regimen, and therapeutic drug monitoring. Mostly administered in neonates as intermittent infusion. Limited data on continuous infusion, but already used in clinical practice.	[55,56,57,58,59,60,61,62,63,64,65]
Vancomycin and ototoxicity	1	1	2	Exposure to vancomycin was not associated with failure of automated auditory brainstem response in neonatal hearing screening. No ototoxicity data on continuous infusion in neonates.	[66,67]
Vancomycin and renal toxicity	1	5	6	Damage due to direct brush border cytotoxic effects. Incidence 11–22%. Not strictly related to specific serum concentrations in children. Elevated trough levels and concomitant furosemide are risk factors. No long-term data on nephrotoxicity in neonates are currently available.	[68,69,70,71,72,73]
Vancomycin for bloodstream/complicated infections	15	15	30	Antibiotic of choice for MRSA infections for more than 4 decades. Widely used for the treatment of neonatal late-onset sepsis, gram-positive invasive infections in children, catheter-related infections, osteomyelitis, pneumonia, septicemia, soft tissue infections endocarditis, central nervous system infections.	[74,75,76,77,78,79,80,81,82,83,84,85,86,87,88,89,90,91,92,93,94,95,96,97,98,99,100,101,102,103]
Vancomycin for *C. difficile* infection	5	5	10	The incidence of CDI has risen in children since 2000. Most pediatric studies have evaluated the incidence of CDI-related hospitalizations among multicenter cohorts of hospitalized children. Highest rates of asymptomatic colonization with either toxigenic or nontoxigenic strains <12 months of age. Oral vancomycin recommended for treatment of pseudomembranous colitis due to *C. difficile*, for the first episode (severe/non-severe; first recurrence; second or subsequent recurrence), 10 mg/kg/dose 4 times daily for 10 days.	[104,105,106,107,108,109,110,111,112,113]
Vancomycin in antibiotic stewardship	1	3	4	Significant decline in vancomycin prescription in hospitalized children with active antibiotic stewardship programs in a multicenter study. Local antibiograms with pathogen-specific susceptibility data should be updated at least annually, to optimize expert-based recommendations for empirical therapy.	[114,115,116,117]
Vancomycin MIC interpretative criteria	2	/	2	Over the last 2 decades, isolates with high but susceptible vancomycin MICs have been associated with additional treatment failures and patient mortality in MRSA bloodstream infections. In 2006, the Clinical Laboratory and Standards Institute reduced the vancomycin-susceptible MIC breakpoint for *S. aureus* from 4 mg/L to 2 mg/L. MIC “creep” as increases over time of central vancomycin MIC tendency.	[118,119]

CoNS: Coagulase-negative Staphylococci; spp: species; MRSA: Methicillin-resistant *S. aureus*; LOS: late-onset sepsis, NICU: neonatal intensive care unit; VISA: vancomycin-intermediate *S aureus*; VRSA; vancomycin-resistant *S. aureus*; IBD inflammatory bowel disease; PK/PD: pharmacokinetics/pharmacodynamics; MIC: minimum inhibitory concentration; VRE: vancomycin-resistant enterococci; HAI: hospital-acquired infections; CDI: *Clostridium difficile* infection.

**Table 3:** Key points on vancomycin use in pediatric patients.

Vancomycin in Pediatric Patients-Key Points	
Spectrum of activity	Bactericidal for aerobic and anaerobic gram-positive bacteria, including coagulase-negative *Staphylococcus* and *S.aureus*. Bacteriostatic for enterococci. Target: Skin and soft tissue infections, bone, and joint infections, bloodstream infections/sepsis/severe infections in children and neonates, endocarditis, nervous system infections. [1] *C.difficile* colitis (oral administration) [108]
Recommended dose—severe infections	Neonates: 10–15 mg/kg (once every 18/12/8 h) [61]
	Children: 60 mg/kg/day in four divided doses [120]
Recommended dose—*C. difficile* colitis	10 mg/kg/dose four times a day [108]
Adverse events	Infusion-related: “red man syndrome”; pain at the injection site; allergic reactions. Drug-related toxicity: neutropenia, thrombocytopenia, eosinophilia, thrombophlebitis, chills, fever, rash, nephrotoxicity, and ototoxicity [2,66,70]
Resistance	VRE: linked to at least 4 genes (Van A-D); selection pressure by extensive use of vancomycin; VISA/VRSA: thickened and aggregated cell walls; S. epidermidis: biofilm [3,4,5,26]
Gaps of knowledge	PK/PD: AUC/MIC ratio; recommended pharmacodynamic target of AUC 24/MIC >400 [1,120,121]
	Continuous infusion (higher chance of target attainment; safety; limit toxicity) [61,62,65,121] vs intermittent infusion
	New dosing strategies in neonates; lack of consensus on dosing regimens in neonates with varying gestational and postnatal ages. Need to dose both for clinical efficacy and to reduce the rate of development of resistance. Efficacy (effectiveness) and safety of increased doses of vancomycin and generic formulations [58,61,62,120].
	Antibiotic stewardship and avoiding further resistances [117]

VRE: Vancomycin-resistant Enterococci; VISA: vancomycin-intermediate *S. aureus*; CA: community-acquired; VRSA: vancomycin-resistant *S. aureus*; PK-PD: pharmacokinetics-pharmacodynamics; AUC: area under the curve; MIC: minimum inhibitory concentration.

## Data Availability

The data presented in this study are available in the Supplementary Material (S1–S3).

## Supplementary Materials

The following are available online at https://www.mdpi.com/article/10.3390/pathogens10101343/s1, Document Supplementary 1: retrieved articles list, Document Supplementary 2: First quartile Infectious Diseases ranked journals list, Document Supplementary3: First quartile Pediatrics ranked journals list.
